# Recent Progress in Printed Photonic Devices: A Brief Review of Materials, Devices, and Applications

**DOI:** 10.3390/polym15153234

**Published:** 2023-07-29

**Authors:** Amal M. Al-Amri

**Affiliations:** Physics Department, Collage of Science & Arts, King Abdulaziz University, Rabigh 25724, Saudi Arabia; amsalamri@kau.edu.sa

**Keywords:** printed photonic devices, printed electronics, optical properties, printing techniques

## Abstract

Printing electronics incorporates several significant technologies, such as semiconductor devices produced by various printing techniques on flexible substrates. With the growing interest in printed electronic devices, new technologies have been developed to make novel devices with inexpensive and large-area printing techniques. This review article focuses on the most recent developments in printed photonic devices. Photonics and optoelectronic systems may now be built utilizing materials with specific optical properties and 3D designs achieved through additive printing. Optical and architected materials that can be printed in their entirety are among the most promising future research topics, as are platforms for multi-material processing and printing technologies that can print enormous volumes at a high resolution while also maintaining a high throughput. Significant advances in innovative printable materials create new opportunities for functional devices to act efficiently, such as wearable sensors, integrated optoelectronics, and consumer electronics. This article provides an overview of printable materials, printing methods, and the uses of printed electronic devices.

## 1. Introduction

Photonic sensors perform remarkable functions and have been extensively utilized in various applications. Flexible and stretchable photonic sensors are necessary to successfully implement emerging technologies, including soft robotics, electronic skin, wearable devices, and flexible displays. These sensors possess the ability to conform to curved surfaces and undergo deformation into various shapes and sizes. Recent years have seen significant advancements in printed photonic devices, opening up novel opportunities for developing low-cost and high-performance optical systems [1,2,3,4]. Printed photonic devices use functional optical components fabricated with advanced printing techniques to create novel customizable devices for a variety of uses [5]. This paper aims to provide a comprehensive review of the progress made in the field of printed photonic devices.

Numerous studies on optoelectronic devices and photonic systems have been carried out on the development of smart image sensors, displays, wearable electronics and screens, neuromorphic devices, and biological healthcare systems [6,7,8,9,10,11]. IoTs have advanced due to the incorporation of printed photonic devices, which have improved connectivity and sensing [10,12]. Energy harvesting [13,14] has also used printed photonic devices, which offer cheap and efficient ways to transform light into electricity.

In the past few years, notable progress has been made in the field of printed photonic devices regarding the development of appropriate materials. Organic semiconductors have garnered considerable attention as a viable option owing to their capacity for customization, ease of processing, and suitability for solution-based printing techniques [15]. Inorganic nanoparticles, such as quantum dots, have exhibited remarkable optical characteristics, facilitating the production of optoelectronic devices with superior performance [16]. The advent of perovskite materials has presented novel opportunities for printed photonic devices, which exhibit exceptional light absorption and emission properties [17]. These devices have a wide range of applications, including but not limited to displays, energy harvesting, and sensing.

Photonic printing based on optical resonators [18,19,20,21,22] has been a topic of extensive research due to its promise as a replacement for existing printing procedures that use chemical pigments. Photonic printing technologies, unlike traditional printing procedures, utilize the geometrical features of a pixelated optical resonator to decide the color of each pixel. Higher picture resolution, material flexibility, great mechanical durability, and improved color fading stability are all possible with this new form of color element. Photon manipulation at the micro/nanoscale is required for both conventional and quantum communication and computation [23,24,25,26]. To enhance optical capabilities over a wide frequency range [27,28,29], a number of materials have been used.

Printed photonics with 2D-material-based inks combines the unique optical properties of 2D materials with a variety of printing techniques to create photonic devices that have the potential to transform a variety of industries, including telecommunications, information technology, sensing, and computing. Written text and graphic works have traditionally been reproduced using printing technologies that allow precise ink placement. Printing techniques have aided in the preservation and transmission of information and knowledge, allowing ideas to survive and contribute to civilization’s growth [30].

To make photonic crystals viable, several technologies have been developed over time. Micro- and nanofabrication for waveguiding plasmon–polariton arrays, electron beams, interference lithography, and molecular beam epitaxy are only some of the techniques that can be used to create porous semiconductors [31]. 3D microstructure printing in soft matrices has the potential to design sophisticated materials with inherent responsiveness [32]. Regular meshes, on the other hand, were only mentioned in a few papers because they were made with a simple technological approach called additive manufacturing. Electromagnetic applications could benefit greatly from the development of photonic crystals that can be 3D-printed and hence easily replicated and deployed. Photonic components made of glassy and soft polymers cannot compete with silicon technology in terms of performance and mass production, at least for the time being [33].

Recently, photonic applications utilizing 4D printing have prompted local configurability (e.g., tunable filters or lasers) [34]. Soft photonic devices entail nanoscale attributes and alternate material integration, whereas robotic and microfluidic responsive systems require “bulky” arrangements with micrometer resolutions (possible by typical lithography process).

Polymer photonics, on the other hand, is a complimentary technique that uses well-designed materials to build arbitrary and customized shapes. Functional materials and their 3D manufacturing play a key part in innovative activities such as photonic device light tenability in this technique. Commercial 3D printers can pattern 3D structures at the macroscale, compared to their microscopic counterparts such as the stereolithography of photocurable resin [35]. Structures can be shrunk to a few microns via laser sintering of polymeric powders [36] or direct ink writing [37,38]. For polymer photonics, direct laser writing (DLW) is chosen because it provides lithographic resolutions of more than 100 nanometers.

The advancement in printing technologies has facilitated the mass production of printed photonic devices. The precise droplet deposition capability of inkjet printing has received considerable attention in the field, enabling the creation of intricate patterns with exceptional resolutions [39]. Screen printing and gravure printing have exhibited their efficacy in printing over large areas and are appropriate for high-volume manufacturing [40]. In addition, advancements in flexographic printing have exhibited the potential for producing flexible and extensible apparatuses, addressing the escalating need for wearable and adaptable electronics [41].

Printed photonic devices comprise diverse optical components, each fulfilling a distinct function. The potential of printed organic light-emitting diodes (OLEDs) for low-cost and energy-efficient displays and lighting applications has garnered significant attention [42]. Using printing techniques to produce photovoltaic devices represents a viable and advantageous approach to achieving the efficient and economical conversion of solar energy on a large scale [43]. Printed technologies have demonstrated promise in facilitating data communication and optical signal routing through waveguides and optical interconnects [44]. In recent times, printed sensors have emerged as powerful instruments for various purposes, such as monitoring the environment and diagnosing healthcare conditions [45].

Nevertheless, the current research and development endeavors, along with advancements in materials, printing technologies, and device architectures, present auspicious pathways for future advancements. Improving the dependability and performance of printed photonic devices is also essential. The subjects of ongoing research are enhancing charge transport properties, minimizing non-radiative recombination, and mitigating material degradation. According to recent studies [46], discovering novel materials with an enhanced charge mobility, stability, and operational lifetime is vital for developing high-performance printed photonic devices.

The commercial viability of printed photonic devices is contingent upon their scalability and mass production capabilities. Important factors include the optimization of the printing process, the enhancement of throughput, and the attainment of uniformity across vast regions. Rosker et al. [47] have investigated automated quality control procedures, continuous manufacturing processes, and roll-to-roll printing as potential enablers for the mass production of printed photonic devices.

Printed photonics is an emerging field with numerous potential applications. 2D materials with good optical properties can be used in various optoelectronic and photonic applications [48]. Cu_2_S, a natural mineral, is used in fluorescent sensors, bioluminescent markers, LEDs, and solar cells. Antimonene, a stable 2D material, is suitable for photonic applications in the UV range. Bismuthine, another 2D material, is considered for inks and printed photonic devices.

Moreover, improved ink formulation methods and the concentration of functional materials are needed to improve efficiency. 2D layered perovskite materials are easy to chemically change and could be used to make efficient 2D photonic devices. 2D printing techniques enable the low-cost, quick, and scalable fabrication of photonic devices with functional inks. These devices exhibit efficient optical properties, revolutionizing fabrication, functions, and layouts. Exploring these properties and their ink formulations leads to unprecedented printed electronic applications.

Future photonic and optoelectronic devices should be compact for high-density on-chip integration, with nanoimprinting being a viable solution for low-cost, high-performance fabrication [49]. Nanoimprinting can process various materials, including thermal plastic polymers, UV-curable resins, and metals, and can be integrated into existing product manufacturing processes. New imprinting resists and process schemes are being investigated, focusing on faster processing speeds, overlay accuracies, and reduced defect generation.

Despite notable advancements, there are still several obstacles that persist in the realm of printed photonic devices. Even with the advancements made in printed photonic devices, challenges related to high-resolution printing, device efficiency and stability, and scalability still need to be solved, impeding their complete potential [50]. Continued research indicates the possibility of discovering resolutions to these issues and advancing the discipline. In this paper, we present the various materials and techniques employed in printing photonic devices by reviewing the literature published in recent years, and discuss the challenges and future opportunities for the fabrication of novel devices with enhanced features.

## 2. Materials for Printed Photonic Devices

**Figure 1:**
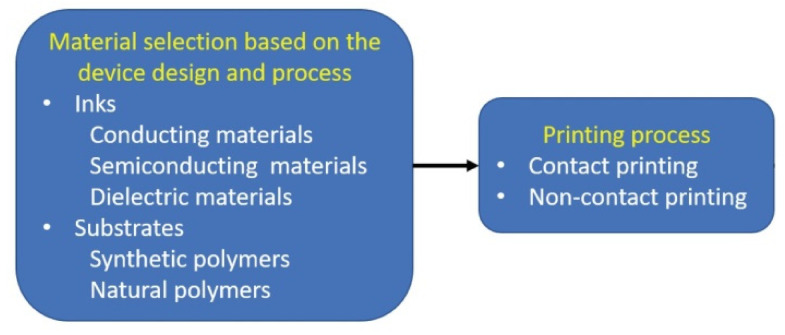
Material Selection and Printing Process [51].

The successful realization of printed photonic devices requires utilizing materials that exhibit advantageous optical, electrical, and mechanical characteristics. The production of printed electronics involves the utilization of primary components such as conductive, semi-conductive, or dielectric inks and substrates that are composed of either synthetic or natural polymers as shown in Figure 1. Significant advancements have been made in the development of purpose-built materials for printed photonic devices in recent times. As a result, the efficacy and efficiency of electronic devices have been enhanced.

### 2.1. Organic Semiconductor Materials

Research is underway to investigate organic semiconductors’ applications in printed photonic devices. According to Rao et al. [52], the materials mentioned in Figure 1 possess various benefits, including their capacity to be processed in solutions, their modifiable band gaps, and their compatibility with a wide range of printing techniques. The significant advancements in OLEDs are a notable demonstration. Tong et al. [53] studied the working principle of OLEDs based on small molecules and polymers that exhibited exceptional luminous efficacy, a diverse spectrum of colors, and flexibility, making them suitable for sophisticated display technologies in the future. Sharma et al. [54] studied the use of organic semiconductor materials in printed photovoltaic devices, particularly organic solar cells. The emergence of affordable, semi-transparent, and flexible organic solar cells (OSCs) has opened up novel opportunities for diverse applications, including building-integrated photovoltaics (BIPs), operating smart wearables and portable electronics, and obtaining indoor lighting for IoT applications.

### 2.2. Inorganic Semiconductor Materials

The application of inorganic nanoparticles in printed photonic devices has attracted considerable interest. Kim et al. [55] have emphasized the potential of nanoparticles in diverse fields due to their exceptional stability, optical properties are dependent on size, and high quantum yields. Quantum dots have exhibited exceptional properties such as limited emission spectra, elevated photoluminescence quantum yields, and the ability to be processed in solution. Low-dimensional nanomaterials, including 0D, 1D, and 2D structures, are used in advanced electronics, optoelectronics, and photonics. They have distinct structural, electronic, mechanical, and optical properties. Their high throughput makes them suitable for large-scale production at a low cost. Photodetectors are essential to modern optical communication and advanced imaging technologies in many areas of daily life, such as biomedical imaging with X-rays, visible light cameras, spectroscopy, and infrared night vision applications.

### 2.3. 2D Materials

Moreover, high-quality inks can be made from 2D materials with unique properties, atomically thin thicknesses, and exceptional solution processabilities [56]. In addition, the wide range of 2D materials, from metals to semiconductors to insulators, allows for the creation of versatile inks for printed electronics. With high-quality functional inks, 2D materials can be obtained in the liquid phase and used in various deposition technologies such as spraying, screen printing, spin coating, 3D printing, and inkjet printing.

Recently, there has been a notable increase in academic research centered on the application of perovskite materials, specifically in printed photonic devices. According to Fukuta et al. [57], hybrid organic–inorganic perovskites demonstrate exceptional optoelectronic properties, including high charge carrier mobilities, prolonged carrier lifetimes, and significant absorption coefficients. Perovskite-based devices have exhibited a commensurate level of efficacy compared to their conventional counterparts. Photovoltaics, LEDs, and other photonic applications stand to benefit the most from the organic–inorganic hybrid lead halide perovskites. The most extensively studied lead halide perovskites have 3D bulk films and crystals, but many 2D hybrid halide perovskites have also been developed. Multilayer structures offer significant design flexibility for the fabrication of novel devices. When metal halide nanosheets and organic materials are stacked on top of each other, they form two-dimensional (2D) perovskites with different electronic systems and optoelectronic properties. The incorporation of heterogeneous materials can lead to the development of hybrid material systems that promise to enhance device functionality and introduce novel attributes. The field of materials science has observed notable growth in the development of sophisticated and versatile photonic devices using printing technology. Moreover, the solution processability and configurable band gap of perovskite materials make them a desirable option for large-scale printing applications [46].

### 2.4. Materials Selection

The successful fabrication of photonic devices using printing techniques is contingent upon the careful choice of materials. Rao et al. [52] have reported that organic semiconductors comprising small molecules and polymers offer several advantages over their inorganic counterparts. Recent studies [54,57] have shown that perovskite materials are promising candidates for solar cells and LEDs due to their outstanding optoelectronic properties. It should be noted that by incorporating two-dimensional materials, such as graphene, and developing hybrid material systems present additional opportunities for improving device performance.

Appropriately and precisely selecting materials is essential to effectively constructing printed photonic devices. Their practical implementation has demonstrated the potential of materials such as organic semiconductors, inorganic nanoparticles, perovskites, and hybrid systems. Additional investigation is necessary within materials engineering to augment the printed photonic apparatus’s efficiency, longevity, and scalability. Table 1 presents a comparison of materials commonly utilized in printed photonic devices. Wearable and flexible polymers possess desirable characteristics such as being lightweight, flexible, and easy to process. Their refractive index range is typically limited, and their temperature stability is moderate. Inorganic materials that lack flexibility exhibit favorable optical characteristics and elevated refractive indices; however, they are prone to brittleness. Hybrid materials are a class of materials that integrate both organic and inorganic characteristics, thereby achieving a harmonious balance between flexibility and optical performance. However, the production of such materials demands a high degree of sophistication. Nanomaterials can be tailored to specific characteristics and exhibit enhanced functionality, albeit at a higher cost and with limited scalability.

The progress in materials technology for printed photonic devices has led to new design, performance, and functionality possibilities. However, there are ongoing difficulties in maintaining the stability of materials, achieving scalability, and ensuring device performance over a prolonged duration, which continue to be a matter of apprehension. Academic researchers are striving to tackle these concerns by creating innovative materials featuring enhanced properties and optimizing printing techniques to guarantee the reliable and uniform fabrication of devices.

In the next section, a comprehensive analysis will be presented regarding the various printing techniques utilized in manufacturing printed photonic devices.

## 3. Printing Techniques for Photonic Devices

**Figure 2:**
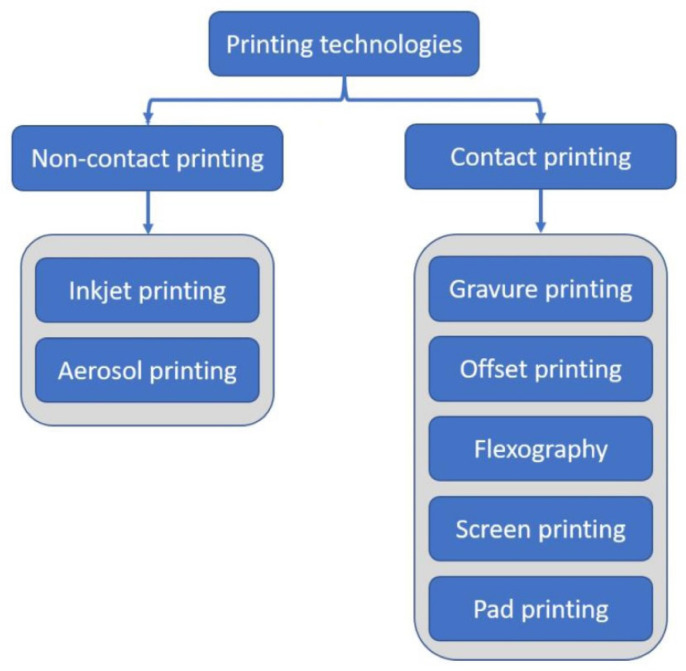
Printing Technology Processes [51].

The selection of a printing technique is a crucial factor in attaining the fabrication of printed photonic devices that are both cost-effective and dependable and achieve a high resolution. Several printing techniques have been investigated, as depicted in Figure 2, each possessing distinct advantages and drawbacks. This section will elaborate upon several unique printing techniques commonly employed within the industry.

### 3.1. Non-Contact Printing Techniques

Non-contact printing methods exhibit high precision and resolution, rendering them well-suited for producing sophisticated photonic structures.

#### 3.1.1. Inkjet Printing

Inkjet printing is a non-contact digital printing technology that produces microstructures as small as 30 microns or smaller. Non-contact printing allows for wet-on-wet application, and the fully digital process control eliminates the need for masks or stencils. Inkjet printing is a highly integrated method where several thousand nozzles can be printed at speeds up to 2 m per second. Inkjet printing replaces traditional subtractive manufacturing methods for electronic devices. By reducing waste and energy consumption, this technology makes the production of electronics more environmentally friendly and, at the same time, more economical. According to Kwon et al. [63] and Ru et al. [64], inkjet printing technology permits precise control over droplet properties, such as size, placement, and density, thereby enabling high-resolution patterning. Yao et al. [65] demonstrated the application of the inkjet-printing method to deposit MXene (2D material) on microring resonator (MRR) waveguides with low loss and a high extinction ratio of 7.8 dB. Using inkjet printing has become increasingly popular due to its low cost, high efficiency, controllability, and scalability. This method has proven to be highly effective in transferring MXene to small-footprint integrated photonic devices. Inkjet printing technology can also be used to produce OLED devices with exceptional efficiency, durability, and color spectrum, as in the case of QD-based devices [66].

**Figure 3:**
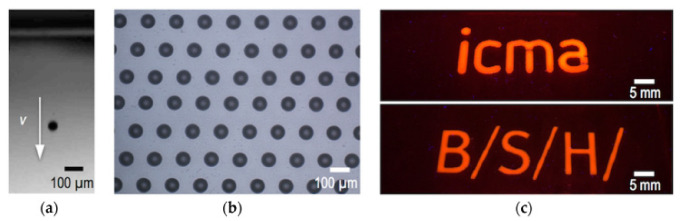
Inkjet-printing process: (**a**) Deposition of picoliter-volume ink droplets at speed v on the substrate; (**b**) Precise deposition of droplets onto a surface; (**c**) Application leads to functional prints or structures [65].

BHJ solar cells have been manufactured on a small scale using this printing technology as well. Inkjet printing can also be used to generate polymeric liquid crystal optical films with real polarization effects, which can be used to preserve papers or objects. As shown in Figure 3, inkjet printing has traditionally been utilized for conventional purposes such as text printing, graphics, and marking due to its capacity to position picoliter volumes of various materials’ with speed (v) under digital control to create innovative functional surfaces and devices. However, nozzle clogging, ink formulation, and substrate compatibility must be addressed to achieve optimal performance.

#### 3.1.2. Aerosol Printing

**Figure 4:**
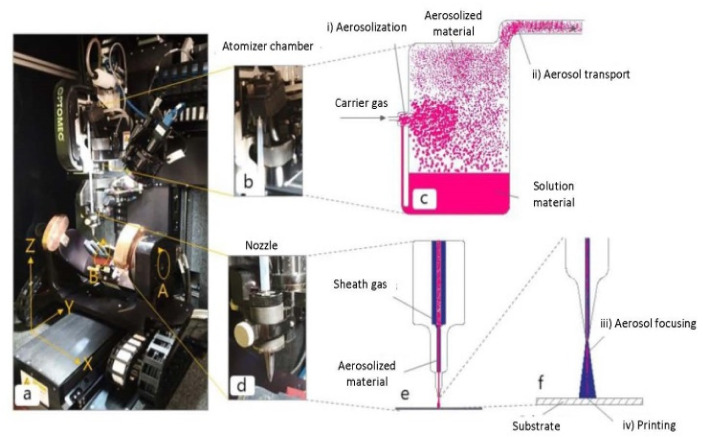
Aerosol jet printing. (**a**) AJ 5X equipment with a 5-axis motion system (Optomec—Albuquerque, USA). (**b**) Atomizer chamber. (**c**) Aerosolization process. (**d**) Aerosol-jet-printing nozzle. (**e**) Aerosolized material and sheath gas. (**f**) Focused aerosolized material [70].

The process of aerosol printing refers to the application of functional materials onto substrates through the deposition of aerosol droplets [67]. This technique provides benefits such as rapid deposition, scalability, and versatility in terms of material compatibility. The utilization of aerosol printing has the potential to attain high-resolution patterns and is deemed appropriate for the deposition of functional materials on large areas. As shown in Figure 4(i), the ultrasonic atomization can aerosolize 2–3 mL of solution into 2–5 μm droplets. The atomization parameters and ink formulation reduce satellite droplets and overspray [1]. In Figure 4, step (ii), a carrier gas (usually nitrogen) transports aerosol to the tip nozzle for controlled droplet diameters [68]. In step (iii), the sheath flow focuses on the aerosol. The optimization parameters involve the ratio of aerosol and sheath flow rates [69]. Step (iv) deposits aerosol over a 2–5 mm range on the substrate from the nozzle tip. However, controlling the droplet size and achieving uniform deposition thickness can be challenging due to satellite droplets [70].

### 3.2. Contact Printing Techniques

In this section, we discuss the following contact printing techniques.

#### 3.2.1. Gravure Printing

**Figure 5:**
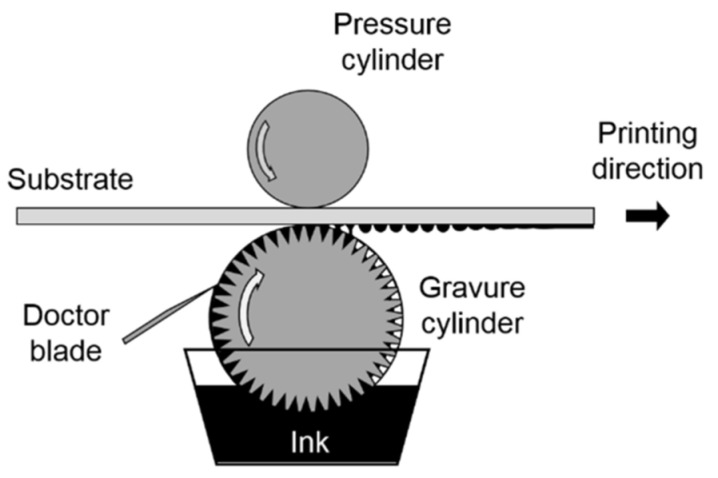
Schematic diagram of roto-gravure printing setup [74].

Gravure printing is one of the most efficient and cost-effective graphic-printing methods. Gravure printing is ideal for printing large, homogeneous regions in polymer electronics, such as typical lighting applications. Gravure printing is a method of obtaining a printed image on paper (or other material) using printing forms on which the printing elements are recessed in relation to the blank (non-printing) elements [71]. The depth of the printing elements on the form varies according to the saturation of the shades of the reproduced image. On paper, this form leaves an impression on which layers of printing ink have different thicknesses, which creates the finest gradations and transitions of tones. Liquid ink is applied to the form’s surface in the printing press, filling the depressions; excess paint from gaps is removed with a special device—a squeegee [72]. The utilization of gravure printing is prevalent in large-scale manufacturing processes owing to its notable capacity for high throughput. Gravure printing is ordered for the high-quality design of catalogs and magazines with illustrations. With its help, unique signs are often applied to labels, stamps, and packaging. This method is preferred because it can print hundreds of meters per minute at a 2 μm resolution. Figure 5 shows how a counter-cylinder’s pressure transfers a low-viscosity ink (1–100 mPa.s) from a chromium-plated cylinder’s micro-engraved cells to a substrate [73]. In this process, doctor blades remove excess ink from un-engraved cylinder surfaces.

This methodology has been employed to produce diverse printed photonic apparatuses, such as organic solar cells, thin-film transistors, and electronic displays. According to Fu et al. [75], the precise management of ink flow and thickness facilitates the creation of high-performance devices that exhibit enhanced device characteristics. However, the printing plate’s fixed pattern constrains its adaptability in generating personalized designs.

#### 3.2.2. Offset Printing

**Figure 6:**
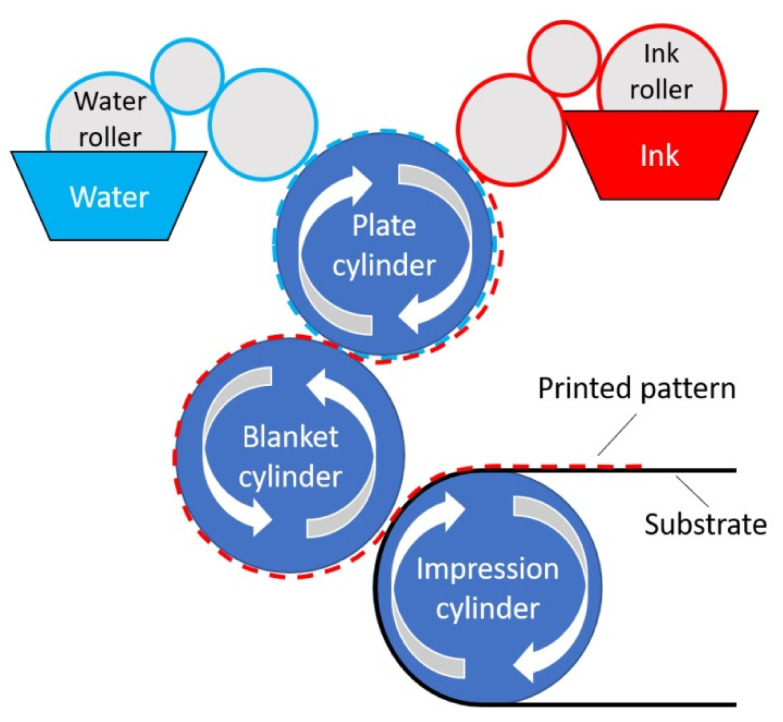
Schematic diagram of offset-printing process [51].

This process employs an indirect printing technique in which ink transfer from a printing plate to the substrate is aided by an intermediate blanket cylinder, as shown in Figure 6. In its classic design, the printing process is carried out on an offset machine, on which two cylinders work and offset. An impression template is placed on the working cylinder, typed from printed elements. During the printing process, during rotation, the template is transferred from the working cylinder to the intermediate one, which in turn transfers the print to the printed surface. An intermediate cylinder allows the user to distribute the printing ink and obtain clearer and better images evenly. Surface chemistry is vital in this process because image parts accept ink while repelling water, while other areas accept water while repelling ink. The variation in surface energy between the image and non-image areas acts as an impediment to ink diffusion. In addition, a thin layer of wetting water in the non-image regions is an additional impediment to the spread of the ink. The ink layer is then transferred under pressure to the blanket cylinder and the paper within the nip. Offset printing uses a wet-on-wet technique to process ink without sequential drying. Thus, the blanket cylinder receives some ink for the next printing unit. The ink layer dries after printing via polymerization, absorption, oxidation, or evaporation, as reported in [76].

Offset printing requires the transfer of ink from a printing plate onto a rubber blanket, which is then used to transfer the ink onto the substrate [77]. The product provides high-quality printing with a high level of resolution, superior color fidelity, and versatility in its compatibility with diverse substrates. Offset printing is prevalent in commercial printing, requiring intricate preparation and thorough calibration.

#### 3.2.3. Flexographic Printing

Flexographic printing is a low-pressure method that employs a photopolymer instead of etched rollers. It uses several methods to print several multilayered device patterns on flexible or rigid substrates. Flexography is a letterpress printing type that uses elastic printing plates and low-viscosity inks. Printing plates are usually made of elastic elastomers. Elevated form elements receive printing ink, and blank elements, deepened by engraving, vulcanization, etching, dissolving, or washing out, remain free of it. Flexography uses quick-drying liquid inks; however, the trend toward using paste inks is increasing. Some types of ink are diluted with solvents, and some with water [78]. The rapid printing speed and compatibility with a diverse range of substrates make it a popular choice for high-volume production. Solution-based deposition and patterning methods such as roll-to-roll (R2R) printing is most suitable for meeting the need for inexpensive, organic, and adaptable electronic components. Flexographic printing provides good registration precision and the capacity to utilize diverse inks, such as functional and conductive inks. This product exhibits rapid printing velocities and elevated ink transfer efficacy and is compatible with various substrates.

**Figure 7:**
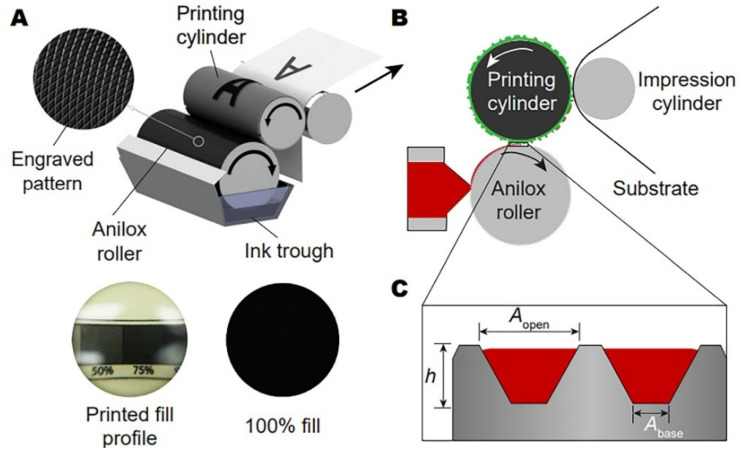
Schematic diagram of a flexographic-printing unit, (**A**–**C**) representing flexographic printing principles [79].

In contrast to gravure printing, flexographic printing employs a relief-printing technique wherein ink is transferred from an elevated surface rather than from carved microcavities. The process of printing can be delineated as follows. Figure 7 depicts a flexographic printing plate, which is both soft and flexible, with raised pixels on the top surface. An anilox roll, whose surface has been etched with small, engraved cells in a uniform pattern, is used to transfer ink to the printing plate’s pixels. The ink from the chamber is spread across the anilox roll, and any excessive ink is removed with a doctor’s blade. The printing plate is then used to transfer the ink to the individual dots. Ink is transferred in a nip where the pressure acting on the ink and the substrate is sufficient to facilitate ink transfer. The ink layer separates at the nip outlet, allowing some ink to flow down onto the substrate below. The printing procedure is completed by transferring the inks from the mold to the drying substrate on the printing cylinder. In a pyramid cell structure, the quantity of ink that a single anilox cell (*V_cell_*) can hold is calculated as follows:

$$V_{cell}=\frac{h}{3}(A_{open}+A_{base}+\sqrt{A_{open}A_{base}})$$

where *h* represents the anilox cell’s height, and *A_open_* and *A_base_* represent the area of the cell opening and base, respectively. This equation varies according to the choice of cell structure. The printing procedure can be customized for various substrates such as carton board, paper, metal, and flexible packaging. The flexographic-printing process can be adjusted to control the pattern length scale and the onset of finger instabilities. This is possible by adjusting the printing velocity, fluid viscosity, and surface tension. Typically, the pattern length scale falls within tens of microns.

Flexographic printing is a widely employed technique for printing on a large scale, particularly in packaging contexts. However, attaining complex attributes and superior resolution can pose a challenge. This technique has produced flexible displays, sensors, and RFID tags within printed photonic devices. Liu et al. [80] suggested that printed electronic (PE) technologies, which combine traditional printing methods with solution-processable functional ink, could be a viable strategy for the low-cost, high-volume production of printed wearable devices.

#### 3.2.4. Screen Printing

**Figure 8:**
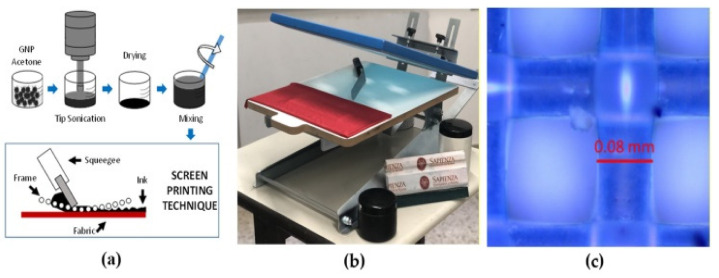
(**a**) Screen-printing fabrication process; (**b**) Screen-printing setup; (**c**) Optical image of fabricated textile wire diameter [82].

The technique of screen printing is extensively employed in the domain of printed electronics, which includes printed photonic devices [81]. The process involves the application of ink onto a substrate using a squeegee through a mesh screen. The mesh screen functions as a stencil, delimiting the design to be printed. Screen printing presents several benefits, including a high throughput, low cost, and the ability to work with different types of inks. This technology exhibits high suitability for printing applications requiring coverage over a large surface area. Marra et al. [82] produced smart textile sensors using the screen-printing technique as shown in Figure 8. The sensors were made from graphene nanoparticle ink that was deposited onto a weft-knitted textile substrate consisting of 96% polyester and 4% elastane fibers, utilizing a manual screen-printing technique. The authors fabricated screen-printed textile with a wire diameter of 80 microns as shown in Figure 8c.

Screen printing has been utilized in producing photodetectors, light-emitting devices, and touch sensors within the domain of printed photonic devices. Using conductive inks through screen printing facilitates the placement of electrodes, interconnects, and active layers, thereby enabling the development of functional devices.

#### 3.2.5. Pad Printing

**Figure 9:**
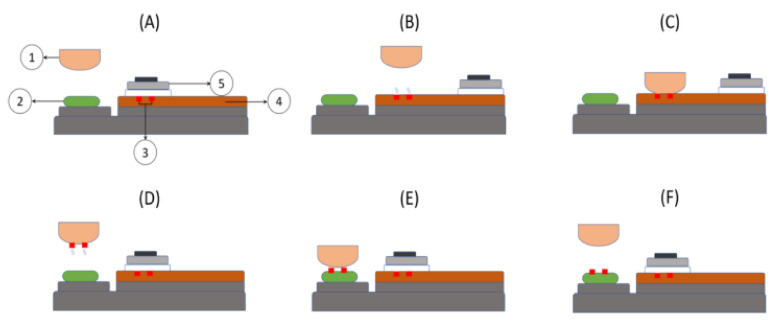
Schematic diagram of pad-printing process (**A**) Rest position with (1) Pad, (2) Substrate, (3) Inked engraved pattern, (4) Cliché and (5) Ink cup. (**B**) Shows the movement of the pad and ink cup at the same time (**C**) patterned ink film is transferred to the pad (**D**) the pad is moved towards the substrate (**E**) the patterned ink film is deposited on the substrate (**F**) the pad moves to its initial position [83].

The pad-printing process involves using a silicone pad to transfer an image onto a substrate. The utility of pad printing lies in its capacity to be employed on a diverse range of irregular surfaces. Pad printing offers a broader range of design possibilities than screen printing and hot stamping. Pad-printing technology can print on diverse materials such as ceramics, glass, plastics, coated substrates, metals, wood, automotive components, pharmaceuticals, cosmetics, food, and other similar substances. The pad-printing process comprises multiple stages or phases as shown in Figure 9 [83]. In step A, the components comprising a resting place are the pad (1), substrate (2), engraved pattern in the ink (3), cliché (4), and ink cup (5). In step B, the ink cup moves to facilitate the evaporation of the solvent present on the upper surface of the engraved pattern. Concurrently, the pad positions itself above the designated region for ink transfer. During step C, the pad acquires the patterned ink film. In step D, the pad experiences a vertical displacement from the substrate and a lateral shift in direction. Currently, the lower surface of the ink film has undergone evaporation of its thinner layer. In order to apply the ink film onto the substrate, the pad descends towards the substrate and establishes contact while the ink cup retracts over the stock pattern to refill it (step D). In step E, the substrate is then elevated and subsequently coated with a layer of ink.

Pad printing involves using a silicone pad to retrieve ink from an etched plate and subsequently transfer it onto the substrate [84]. This technology provides a range of applications due to its versatile nature, high-resolution printing capabilities, and capacity to print on non-uniform surfaces. The pad-printing technique is frequently employed in manufacturing three-dimensional items on a small scale. Attaining accurate alignment and uniform ink distribution can pose a difficulty.

This section comprehensively discussed various distinguished printing techniques for producing printed photonic devices, and a comparison is shown in Table 2. The selection of a printing methodology is contingent upon many factors, such as the intended resolution, the substrate’s flexibility, and the materials’ compatibility. Various techniques present distinct benefits and drawbacks, and researchers continue to investigate and create innovative printing techniques to cater to particular demands in the field of printed photonic devices.

## 4. Device Components and Their Use in Printed Photonic Devices

This section will discuss the primary components typically used in printed photonic devices and their integration strategies.

### 4.1. Light-Emitting Device Components

Light-emitting devices, including organic light-emitting diodes (OLEDs) and quantum dot light-emitting diodes (QLEDs), play a pivotal role in various fields, such as graphical displays, lighting, and directional signs [89,90]. The fabrication of components in printed photonic devices is commonly achieved through solution-based techniques, followed by careful patterning and layer-by-layer assembly to integrate them into the device structure [91,92].

**Figure 10:**
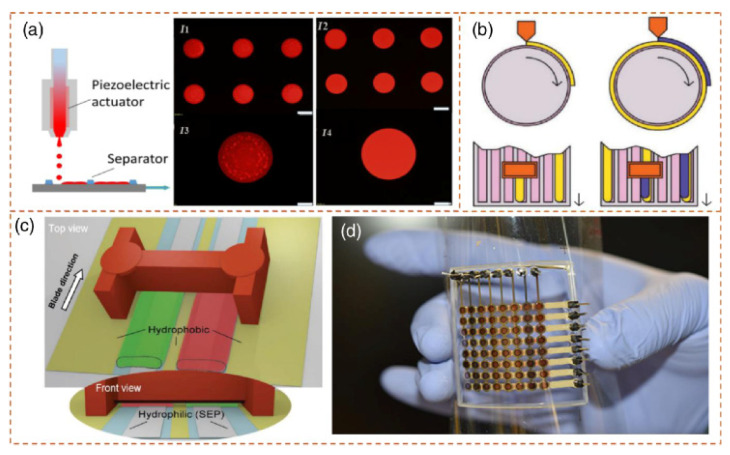
(**a**) Schematic of inkjet-printing process with separator. I_1_ and I_2_ represent fluorescence microscopy imaging of the QD array with different concentrations, whereas higher-resolution images corresponding to single dots are shown as I_3_ and I_4_. (**b**) Schematic view of slot-die-roll-coating process. (**c**) Blade-coating process (top and front view) for fabricating OLEDs (multicolor). (**d**) Illustration of the final 3D-printed OLED display [93].

The light-emitting layers in OLEDs are comprised of organic semiconductors that can emit light upon applying an electric current. The process of incorporating light-emitting components into printed photonic devices entails the application of organic layers onto the appropriate substrates. According to [93,94], precise layer thickness and uniformity can be attained through inkjet-printing, slot-die-coating, and blade-coating techniques. A multicolor OLED depicted in Figure 10 was fabricated on a flexible substrate [93]. As shown in Figure 10a, the thickness of the film was controlled by varying the concentration of ink and the drop rates within a single pixel. The selection of materials and deposition methodologies is contingent upon various factors, including but not limited to the performance specifications of the device, the use in large-area, flexible organic semiconductor devices, and the sustainability and recyclability features with reduced fabrication cost. Moreover, quantum dot nanoscale semiconductor particles with adjustable emission properties constitute the emissive layers of QLEDs. The accurate management of the placement and design of quantum dots is imperative for attaining consistent and high-quality emission in quantum dot light-emitting diode (QLED) technology.

### 4.2. Photodetectors and Sensors

Photodetectors and sensors are essential components in the detection and capture of light signals across a range of applications, such as communication systems, sensing, and imaging. Photodetectors and sensors are commonly incorporated into the architecture of printed photonic devices through the utilization of semiconducting materials during fabrication.

**Figure 11:**
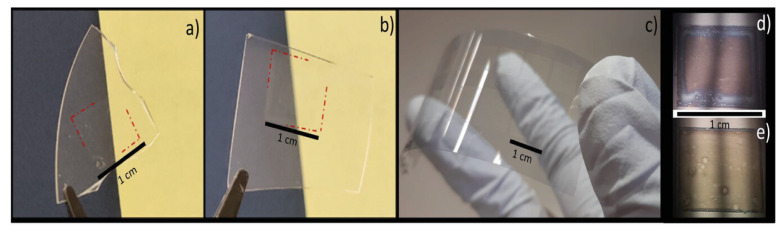
Inkjet-printed layers on fused silica substrates. (**a**) NiO and (**b**) ZnO (**c**) layers printed on flexible substrates. Close-up view of (**d**) NiO and (**e**) ZnO [95].

An NiO/ZnO heterojunction as shown in Figure 11, thin-film photodetector was made using an inkjet printing technique in ambient conditions [95]. Individual layers of high-quality NiO and ZnO were made with no surface defects and excellent transparency in the visible range. The device showed clear rectifying behavior in the dark, and a wide-band photoresponse when exposed to AM1.5 Global sun simulator light. It is possible to make optoelectronic devices using scalable, no-mask, inkjet-printing technology. The devices were printed on fused silica, which lets more UV light through than standard glass, and on flexible substrates, showing high-quality layers in both cases.

**Figure 12:**
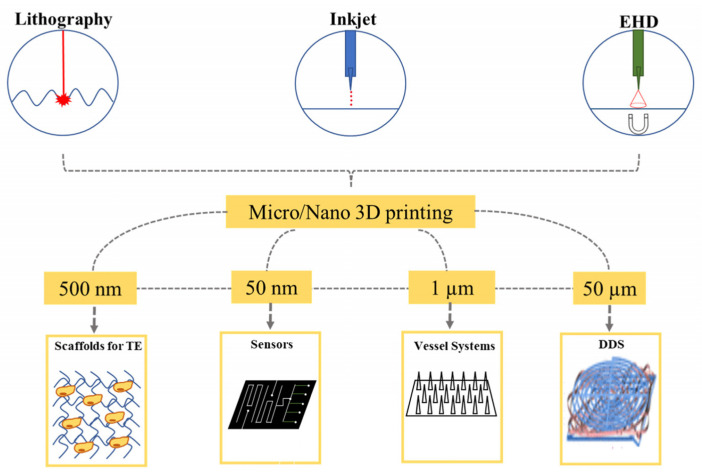
Various printing processes at micro/nanoscale for 3D printing of electronic devices [96].

A comprehensive overview of the micro/nano-printing techniques of lithography, inkjet, and EHD printing is shown in Figure 12 [96]. By incorporating micro/nanostructure materials, the printing technique can transform materials’ physical and chemical properties in a way that cannot be accomplished with conventional fabrication methods. Printed photonic devices can incorporate specialized sensors, such as gas or biosensors and photodetectors. Sensing elements frequently comprise functional materials, including nanomaterials or conductive polymers [97]. Various printing methods, such as inkjet printing and screen printing, enable the targeted placement of sensing materials onto the appropriate substrates, thereby enabling the incorporation of sensors into the device architecture [98].

### 4.3. Waveguides and Optical Components

Waveguides and optical components play a pivotal role in integrated photonic systems by facilitating the routing and modification of light signals [99]. Various materials and printing techniques can be employed to fabricate waveguides and optical components in printed photonic devices [100].

Polymer-based waveguides are prevalent in printed photonic devices due to their flexibility and easy manufacturing process [101]. The deposition of waveguide materials onto substrates can be achieved through inkjet-printing or screen-printing techniques. According to Alamán et al. [102], these materials may include polymers or hybrid organic–inorganic materials. The precise printing of waveguide structures, followed by encapsulation with the appropriate materials, can accomplish the patterning and integration of waveguides.

Printed photonic devices can incorporate optical components, including gratings, lenses, and filters, to effectively control light signals. According to Raut et al. [103], using printing methods such as inkjet printing and flexographic printing facilitates the application of functional materials onto substrates, thereby producing said components. The optimal optical characteristics and performance of devices are dependent upon the design and patterning of their components [104].

The selection of materials for waveguides and optical components is dependent upon various factors, including but not limited to the refractive index, transparency, and suitability for printing techniques [105]. The optical properties and printability of various materials, such as polymers, glass, and hybrid organic–inorganic materials, have been investigated [106]. It is crucial to consider surface treatments and the engineering of interface properties to reduce optical losses and improve light confinement in waveguide structures [107].

The successful combination of waveguides and optical components in printed photonic devices necessitates accurate alignment and coupling with other device elements, including light sources, detectors, or optical fibers [108]. It is suggested that sophisticated alignment techniques, such as vision-based systems [109] and automated alignment algorithms [110], can effectively attain precise alignment and optimize optical performance [111]. Furthermore, optimizing coupling interfaces and implementing effective light-coupling strategies are imperative to attaining elevated transmission efficiency and mitigating losses [112].

### 4.4. Device Characterization and Performance Evaluation

Various characterization techniques are used to identify crucial device parameters such as optical properties, electrical characteristics, and device responses. Optical characterization techniques such as spectroscopy, ellipsometry, and near-field imaging are used to gauge the optical characteristics of printed photonic devices [113]. These techniques offer details on parameters such as light absorption, emission spectra, waveguide propagation characteristics, and refractive indices. The reliability and stability of printed photonic devices must also be assessed based on how well they perform under various operating conditions, such as temperature, humidity, or mechanical stress [114].

The manufacturing process of printed electronic (PE) devices is undergoing rapid evolution to integrate high-speed printing approaches. Nevertheless, the techniques necessary to achieve high-speed production while preserving superior quality standards continue to pose significant challenges. The primary concern pertains to integrating active, real-time feedback into the process. The integration of all-optical difference engine (AODE) sensors and terahertz time-domain spectroscopy (THz-TDS) quality control methods can offer a comprehensive analysis of defects and the PE production process, as stated in reference [115]. A production process can acquire knowledge and improve itself autonomously by utilizing artificial intelligence (AI) assistance, such as machine-learning algorithms (MLAs). The creation of a printed electronics production database could permit the continuous enhancement of device performance while reducing production costs.

The growing demand for flexible electronics in various consumer applications has prompted the research community to seek cost-effective solutions with high-performance capabilities. Flexible electronics have many possibilities due to advances in information and communication technology, sensors, and actuators for IoT and Industry 4.0 devices. Integrating organic and inorganic materials can lead to expedited and effective results in producing hybrid integrated, flexible electronics, as they work in tandem to complement each other. The process can utilize traditional, inflexible surface-mounted components and flexible, highly slim, bare silicon chips. Palavesan et al. [116] presented the roll-to-roll (R2R) manufacturing process of electronic devices in detail and included a case study on creating radio frequency identification tags. The article also investigated the processes of the R2R manufacturing of metal wiring lines on films and hybrid integration. However, film-based flexible electronics only benefit when hybrid integration technology is transferred to a continuous R2R process. A continuous R2R production can be evolved by additive processes, printing, and self-assembly [117]. New quality control and metrology forms are needed to store, monitor, and analyze real-time production data [118]. Process drift can be corrected in real time using artificial intelligence and other deep-learning techniques [119]. Future inline metrology will increasingly incorporate techniques derived from big data analysis. They provide instant morphology for a wide variety of materials and products, with a spatial resolution on the order of 10 μm and requiring minimal calibration [118]. It has been reported that eddy current sensors are the best choice for noncontact inline R2R electrode quality monitoring [120]. For lab-scale inline R2R measurement, spectroscopic ellipsometry, hyperspectral single point, imaging reflectometry, and Raman spectroscopy are being developed. Polarimetry, digital holography microscopy, and optical coherence tomography are promising methods for inline R2R metrology [118].

## 5. Applications of Printed Photonic Devices

The development of printed photonic devices has shown attractive prospects for diverse applications across various fields. This section examines the emerging application domains that significantly impact printed photonic devices.

### 5.1. Printed Photonic Integrated Circuits (PICs)

As an optical analog of an electrical loop, a photonic integrated circuit (PIC) can process signals at a rapid rate and simultaneously. In order for PICs to work properly, a variety of materials must be employed in conjunction with silicon-based circuitry. In the same way that flexible electronics can be employed, PICs and optoelectronic components can be combined on a single chip [121].

Optimizing the preparation conditions and device geometries could improve the performance of the printed PICs described here. Improved microstructure control can be achieved using the same multi-nozzle technology as a color printer. A single chip with incorporated light-filtering and storage units showed the organic printed photonics’ capacity to process light signals. To enhance the performance of these printed PICs, more complex integrations with different doping materials might be produced simultaneously [122,123].

### 5.2. Wearable Electronics and Smart Textiles

**Figure 13:**
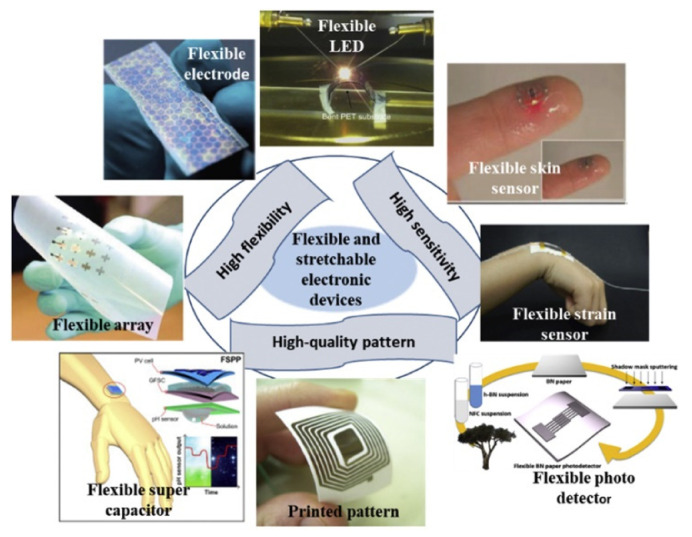
Printed electronic devices for various applications [124].

The utilization of printed photonic devices in wearable electronics and smart textiles has sparked considerable interest in the scientific community. Because of their adaptability and flexibility, printed devices are ideal for incorporation into clothing, gadgets, and other wearable items. Wearable electronics are becoming increasingly important in the electronics market. The demand for printed wearable electronic applications has recently increased. This requires the discovery of novel and easily processed materials to pave the way for developing wearable devices with high-quality electronic properties. Various carbon and metal nanomaterials and conductive polymers are being investigated as potential materials for the fabrication of flexible, adaptable, and wearable printed electronic devices. These devices can perform a variety of functions, including health monitoring, gesture recognition, and customized lighting as shown in Figure 13 [124].

Flexible and printed sensors have the potential to be integrated into garments to monitor vital physiological parameters such as heart rate, body temperature, and respiratory rate [125]. Remote health monitoring using non-invasive and wearable sensors, actuators, and modern communication and information technologies allows patients to stay home instead of using expensive healthcare facilities. These systems will also allow healthcare workers to monitor vital signs, assess health, and provide feedback from remote facilities. Hence, integrating sensors into the fabric makes it possible to achieve non-invasive monitoring of an individual’s health parameters [126].

The development of intelligent luminous textiles has garnered significant attention due to their potential applications in various fields, including but not limited to clothing, interior design, and visual merchandising. In addition, using luminous textiles is advantageous in protective clothing and sportswear as it enhances safety through increased visibility and interactive design, thereby facilitating non-verbal communication. Furthermore, the potential of luminous textiles in healthcare and medicinal applications, such as phototherapy, is significant. Hence, incorporating printed light-emitting devices into textiles has the potential to create interactive garments or provide visual feedback in response to environmental stimuli or gestures [127].

### 5.3. Internet of Things (IoT) and Smart Environments

The adoption of printed photonic devices is significant for advancing Internet of Things (IoT) applications and intelligent environments [128]. The integration of gadgets, such as communication components, sensors, and actuators, into multiple objects and surroundings facilitates the gathering and transmission of information for intelligent and interconnected systems.

Printed sensors offer major advantages over other sensing technologies: they can be prototyped and manufactured at scale, they are disposable, low-power, flexible, stretchable, and small, and they can be integrated into IoT networks. The utilization of printed sensors and photodetectors has the potential to facilitate environmental monitoring, specifically in air quality sensing and light intensity detection [129]. The cost-effective and easily expandable characteristics of printing techniques render it viable to implement a vast array of dispersed sensors for thorough environmental surveillance.

Another study presented a flexible, wireless LED patch with an Internet of Things (IoT) healthcare scheme for injury recovery. The flexible LED patch had good device uniformity, thermal stability, and mechanical stability for skin-attachable phototherapies. Integrating an IoT-connected healthcare system with a smartphone application for the flexible LED patch presents significant opportunities for developing a cost-effective remote healthcare system. In addition, there was a substantial correlation between wavelength and exposure duration in regard to biological responses and migration effects. The optimization of LED wavelengths and radiation doses is necessary for improved clinical assessment. The findings showed that the flexible LED patch exhibited promising potential as a photomedical device for dermatological applications [130].

In addition, using printed photonic devices has the potential to facilitate the development of intelligent lighting systems that offer improved regulation and energy conservation [131]. Light-emitting devices that are printed, such as organic light-emitting diodes (OLEDs) or quantum dot light-emitting diodes (QLEDs), have the potential to be incorporated into lighting fixtures. This integration can offer the benefits of tunable colors, adjustable brightness, and efficient energy consumption. These technological devices can provide customized lighting settings, adaptable illumination, and incorporation with Internet of Things (IoT) platforms to facilitate intelligent lighting management.

### 5.4. Biomedical and Healthcare Applications

The progress in printed photonic devices has benefited the biomedical and healthcare sectors. These devices can be diagnostic, drug administration, and continuous health monitoring tools through wearable technology.

The integration of organic and printed electronics holds promise for transforming various technologies, such as biomedical diagnostics. Ahmadraji et al. [132] showcased the effective combination of diverse printed photonic electronic functionalities within a single device that had the ability to measure both hydrogen peroxide and total cholesterol levels. The employed device was a disposable device that utilized printed electrochemical sensors. Printed sensors and biosensors are also utilized in diagnostics to identify a range of analytes, including lactate, glucose, and other common and specific biomarkers [133]. The integration of these sensors into portable devices or wearable platforms can enable swift and non-intrusive health monitoring. Various physical sensors have been developed to monitor basic biometric parameters using physical sensing mechanisms. These measurements include skin temperature, human motions by strain sensors, the detection of human pulse waves by pressure sensors, and measuring electrocardiogram signals. Moreover, physical sensing platforms with multiple functions have been designed using advanced materials and studied extensively [134]. Moreover, printed photonic devices can be used in point-of-care testing, thereby furnishing economical and easily accessible diagnostic instruments in settings with limited resources.

Printed photonic devices are known to significantly contribute to drug delivery systems, particularly in cases where the precise control and targeted release of therapeutic agents are of the utmost importance [135]. 3D printing and additive manufacturing have transformed personalized drug delivery systems. Additive manufacturing allows customized drug products with a high dose, shape, and size flexibility to meet patient needs [136]. 3D printing can make patient-specific drug delivery devices with high customization, low production costs, and low unit-to-unit variability. 3D printing helps create therapeutic drug delivery systems and biomedical artificial tissues and organs. Razavi et al. [137] have demonstrated that printing techniques can be utilized to fabricate microfluidic devices that incorporate optical components. This approach enables accurate manipulation of fluids and regulated drug delivery. The incorporation of light-sensitive materials or structures that respond to stimuli in printed drug delivery systems facilitates the release of drugs as needed or the initiation of therapeutic responses [138].

### 5.5. Energy Harvesting and Sustainable Technologies

Printing techniques provide a cost-effective and scalable method for producing solar cells and energy-harvesting devices, which can be utilized for renewable energy generation. Photovoltaic devices, including organic and perovskite solar cells, can be incorporated into building structures, windows, or portable chargers to convert sunlight into energy [139]. High-resolution printing techniques have also enabled the flexible and large-area fabrication of photovoltaics. Printed solar cells are a viable alternative to silicon-based solar panels due to their lightweight and flexible properties, making them suitable for applications where traditional panels are impractical.

The integration of solar cells into portable electronic devices and consumer products imposes strict requirements in terms of the weight, mechanical properties, and cost of PV devices [140,141]. Printed energy storage devices such as supercapacitors or batteries provide advanced sustainable energy solutions [142,143]. Printed energy storage devices provide benefits such as rapid charging, lightweight construction, and suitability for use with flexible and portable electronic devices for harvesting renewable energy.

## 6. Challenges and Future Perspectives for Printed Photonic Devices

Printed photonic devices have excellent potential for future advancements and widespread adoption in various industries. However, many challenges must be overcome to realize their potential. The integration of printed photonic devices presents a number of obstacles that must be overcome to guarantee performance and functionality. This section will examine some of the most significant integration challenges, their prospects, and the strategies to overcome them.

### 6.1. Alignment and Registration

The performance of a printed photonic device greatly benefits from careful alignment and registration of its various components [144]. Optical losses, decreased device efficiency, and compromised functionality are all possible results of improper alignment [145,146]. Conventional fabrication techniques such as lithography rely on precise positioning and alignment markers to achieve alignment. However, alignment becomes more complex in printed photonic devices because layers are deposited using printing techniques [147].

One way to deal with alignment and registration issues is to use alignment marks and fiducial markers in the printing process [148,149,150]. In subsequent printing steps, these markers can be reference points for accurate layer and component alignment [150]. Alignment processes may result in more precise and time-efficient devices through automated alignment methods and state-of-the-art vision systems.

### 6.2. Material Compatibility and Interface Engineering

The advancement of printed photonic devices requires the creation of novel materials that exhibit improved optical, electrical, and mechanical characteristics. Moreover, the compatibility of materials is a crucial factor to be taken into account in the integration of printed photonic devices. The proper functioning and long-term stability of various components, including waveguides, light-emitting layers, and photodetectors should be made compatible with the materials used for each component. Furthermore, the interface design between distinct materials should reduce interfacial defects, augment charge transfer, and optimize optical coupling.

The utilization of interface engineering methods, including interfacial modifiers, surface treatments, and interlayer materials, has been demonstrated to enhance device performance and improve material compatibility [151]. Various techniques for surface functionalization, such as plasma treatment and self-assembled monolayers, have been developed to alter the surface characteristics of materials, thereby enhancing their adhesion and compatibility. Moreover, implementing interlayer materials that possess customized properties can function as buffer layers, thereby reducing interfacial strain and augmenting the efficacy of charge transport [152,153,154,155,156,157,158].

### 6.3. Device Reliability and Stability

For practical applications, printed photonic devices’ long-term reliability and stability are vital. The performance of these devices can be affected by environmental conditions, aging effects, and material degradation, among other variables [159,160]. Researchers should concentrate on developing stable materials, robust fabrication processes, and dependable encapsulation techniques to increase the devices’ reliability and lifespan [161].

Implementing encapsulation methods, such as barrier films or protective coatings, can safeguard printed photonic devices against external elements, including moisture, oxygen, and UV radiation [162,163]. The implementation of encapsulation layers can serve as a safeguarding mechanism, impeding the deterioration of delicate constituents and augmenting the overall stability of the device [164,165]. Furthermore, implementing sophisticated packaging methods, such as flexible packaging and hermetic sealing, can augment the dependability and longevity of printed photonic components [166].

### 6.4. Scalability and Manufacturing Processes

The ability to scale fabrication processes is a crucial factor in the successful commercialization and widespread adoption of printed photonic devices. Printing techniques provide cost-effective and flexible substrate-compatible advantages; however, their scalability can pose certain difficulties. Reducing production costs and allowing for mass production requires the optimization of printing processes, the development of cost-effective materials, and the establishment of large-scale manufacturing methods.

In order to tackle the issue of scalability, researchers have established roll-to-roll (R2R) manufacturing techniques, which enable the seamless execution of printing and patterning on a flexible substrate roll in a continuous manner [167,168]. The utilization of R2R printing has the potential to enhance production throughput and diminish manufacturing expenses substantially [169]. In addition, the advancement of high-velocity printing mechanisms [170], the enhancement of ink compositions [171], and upgrades in printing machinery can facilitate the mass production of printed photonic apparatus [172].

## 7. Conclusions

In summary, the utilization of printed photonic devices has emerged as a promising technology with a diverse array of potential applications. The advancements in materials, printing techniques, and device fusion have facilitated the development of photonic systems that are cost-efficient, adaptable, and expandable. Printed photonic devices provide distinct benefits and introduce novel prospects for diverse sectors, encompassing displays, sensors, energy harvesting, and lighting.

Although notable advancements have been achieved, several issues require attention, including printing resolution, material efficacy, device dependability, scalability, and system integration. In conjunction with collaborative initiatives between academia and industry, sustained research and development initiatives are imperative in tackling these limitations and exploring the potential of printed photonic devices.

As the field advances, it is crucial to prioritize investigations on innovative materials that exhibit superior optical and electrical characteristics and augmented mechanical flexibility. The investigation of novel synthesis methodologies and the enhancement of material compositions have the potential to yield materials with expanded functional capabilities and enhanced performance attributes. In addition, it is imperative to enhance printing procedures to attain superior resolution, accuracy, and productivity. This may involve the exploration of novel printing techniques, the enhancement of ink compositions, and the refinement of print head architectures. The progression of printing technology is expected to facilitate the production of complex photonic structures with higher resolution, resulting in enhanced device efficacy and broader application potential.

Moreover, it is imperative to tackle the issues pertaining to device dependability, sustained stability over an extended period, and resilience in various environmental conditions. This includes encapsulation methodologies that safeguard the printed photonic apparatus against environmental factors such as humidity, thermal fluctuations, and physical strain. The successful commercialization and widespread adoption of printed photonic devices are contingent upon the assurance of their reliability and long-term performance.

Future research should focus on the production of high-quality, low-cost, environmentally friendly stretchable and wearable electronics on a large scale. To maintain their functionality as sensor components, conductive inks must meet several challenges, including good dispersion, fluid properties, adhesion to substrates, post-processing by annealing at low temperatures, and minimization of toxic solvents. It is necessary to investigate and optimize ink formulations for emerging printing technologies.

The practical implementation of printed photonic devices necessitates crucial manufacturing scalability and cost-effectiveness considerations. For the printing process, high-resolution and high-performance printing techniques must be considered. R2R production is a common industrial process for mass production and high performance, and the rapid development of 3D printing methods for producing flexible electronic devices. Researchers must investigate various techniques for large-scale manufacturing to facilitate the mass production of printed photonic devices while mitigating expenses. The integration of these devices into diverse industries and applications, such as wearable electronics, healthcare monitoring, communication systems, and smart infrastructure, will be facilitated.

## Figures and Tables

**Table 1:** Comparison of Materials for Printed Photonic Devices.

Materials	Advantages	Limitations	References
Polymers	(i) Easy to process (ii) Lightweight and low cost (iii) Flexible	(i) Low conductivity (ii) Moderate thermal stability	[58,59]
Inorganic	(i) Excellent optical properties (ii) High refractive index	(i) Limited flexibility (ii) Expensive	[60]
Hybrid	(i) Flexibility and conformability (ii) Multifunctionality (iii) Enhanced light interaction	(i) Optimization challenges (ii) Complex fabrication	[61]
Nanomaterials	(i) Enhanced functionality (ii) Tunable properties	(i) Limited scalability (ii) High cost	[62]

**Table 2:** Comparison Table: Printing Techniques for Printed Photonic Devices.

Printing Technique	Scalability	Advantages	Applications	Printing Speed	Line Width/Thickness	Limitations	References
Inkjet Printing	Scalable	Compatible with various materials, control of droplet size with precision.	Various	Moderate to High	30–50 µm/ ~1 µm	Clogging, ink formulation challenges, and issues with substrate compatibility.	[85]
Aerosol Printing	Scalable	Scalable and compatible with various materials in high-speed deposition requirements.	Large-area deposition	Low	20–40 µm/ ~30 nm	Challenges in achieving uniform deposition thickness and precise control of droplet size.	[86]
Gravure Printing	High	Good reproducibility and ink transfer efficiency. Precise deposition with high speed.	Mass production	High	10–50 µm/ ~1 µm	Limited flexibility due to fixed pattern. Expensive plate fabrication mechanism.	[87]
Offset Printing	High	Excellent color reproduction and applicable to a variety of substrates.	Commercial printing	High	50–170 µm/ ~2 µm	Complex setup and expensive, inability to quickly make adjustments.	[77]
Flexographic Printing	High	High ink transfer efficiency, fast printing speeds, and appropriate for many materials and substrates.	Packaging, large area	High	45–100 µm/ <1 µm	Challenges in attaining high resolution, limited ink transfer consistency on irregular surfaces.	[88]
Screen Printing	Moderate	Simple and Versatile. High throughput. Can be used for a variety of substrates. Low cost.	Displays, sensors	Moderate	30–50 µm/ 5–100 µm	Limited resolution due to mesh size constraints. Restricted to mostly thick film depositions.	[81]
Pad Printing	Moderate	Versatile and able to print on irregular surfaces.	Three-dimensional objects	Moderate	100–150 µm/ 0.1–0.15 mm	Challenges in consistent ink transfer.	[83]

## Data Availability

No primary data was used for the research described in the article.
